# Recent Progress of Cellulose-Based Hydrogel Photocatalysts and Their Applications

**DOI:** 10.3390/gels8050270

**Published:** 2022-04-26

**Authors:** Jinyu Yang, Dongliang Liu, Xiaofang Song, Yuan Zhao, Yayang Wang, Lu Rao, Lili Fu, Zhijun Wang, Xiaojie Yang, Yuesheng Li, Yi Liu

**Affiliations:** 1 Hubei Key Laboratory of Radiation Chemistry and Functional Materials, Non-Power Nuclear Technology Collaborative Innovation Center, Hubei University of Science and Technology, Xianning 437100, China; yjyxjj@wust.edu.cn (J.Y.); ldl142325@163.com (D.L.); yuebanwan68@163.com (X.S.); zhyf308@hbust.edu.cn (Y.Z.); wyy1750934313@163.com (Y.W.); rl13908440364@163.com (L.R.); fll151852538462022@163.com (L.F.); qwertasdkl@163.com (Z.W.); mailyangxiaojie@126.com (X.Y.); 2 Key Laboratory of Coal Conversion and New Carbon Materials of Hubei Province, School of Chemistry and Chemical Engineering, Wuhan University of Science and Technology, Wuhan 430081, China; 3 College of Chemistry and Chemical Engineering, Tiangong University, Tianjin 300387, China

**Keywords:** cellulose, cellulose derivatives, hydrogels, photocatalytic composites

## Abstract

With the development of science and technology, photocatalytic technology is of great interest. Nanosized photocatalysts are easy to agglomerate in an aqueous solution, which is unfavorable for recycling. Therefore, hydrogel-based photocatalytic composites were born. Compared with other photocatalytic carriers, hydrogels have a three-dimensional network structure, high water absorption, and a controllable shape. Meanwhile, the high permeability of these composites is an effective way to promote photocatalysis technology by inhibiting nanoparticle photo corrosion, while significantly ensuring the catalytic activity of the photocatalysts. With the growing energy crisis and limited reserves of traditional energy sources such as oil, the attention of researchers was drawn to natural polymers. Like almost all abundant natural polymer compounds in the world, cellulose has the advantages of non-toxicity, degradability, and biocompatibility. It is used as a class of reproducible crude material for the preparation of hydrogel photocatalytic composites. The network structure and high hydroxyl active sites of cellulose-based hydrogels improve the adsorption performance of catalysts and avoid nanoparticle collisions, indirectly enhancing their photocatalytic performance. In this paper, we sum up the current research progress of cellulose-based hydrogels. After briefly discussing the properties and preparation methods of cellulose and its descendant hydrogels, we explore the effects of hydrogels on photocatalytic properties. Next, the cellulose-based hydrogel photocatalytic composites are classified according to the type of catalyst, and the research progress in different fields is reviewed. Finally, the challenges they will face are summarized, and the development trends are prospected.

## 1. Introduction

With the economy’s progress and the improvement in living standards, environmental and energy problems are becoming increasingly prominent. The emission of dyes, heavy metals, pesticides, and the emergence of a vast number of microbial pathogens is extremely hazardous to human health and can even cause system disorders, cancer, and other diseases [1,2]. Researchers have carried out the disposal of organic pollutants and heavy metal ions by employing adsorption [3], reverse osmosis [4], and ion exchange [5]. Still, these techniques have limitations, such as high cost and low efficiency. For the threat of pathogenic bacteria, antibiotics [6], ultraviolet light, and high-temperature sterilization [7] are commonly used. However, antibiotic sterilization tends to cause the development of drug-resistant strains, and UV and high-temperature sterilization also have limitations. Meanwhile, energy shortages affect socioeconomic development and human living standards. Therefore, it is urgent to seek a green, safe, and sustainable energy technology. Photocatalytic technology uses sunlight to irradiate photocatalytic materials in order to degrade organic pollutants, reduce heavy metal ions, inactivate bacteria, and produce hydrogen, which is highly efficient, green, safe, and cheap, and is one of the ideal ways to solve environmental and energy problems.

Nanosized photocatalysts are easily agglomerated in water and are not suitable for recovery. Activated carbon [8], molecular sieve [9], hydrogel [10], and other materials are usually used as carriers to immobilize photocatalysts and improve the utilization rate. Hydrogels, with high permeability, adsorption, and insolubility, are a type of bionic photocatalytic reactor that has received wide attention from researchers [11]. Hydrogels are three-dimensional network structures formed by electrostatic interaction, an entanglement of molecular chains, and cross-linking of chemical bonds [12]. Due to their absorption of large amounts of water and certain flexibility, they can form various hydrogels, such as gel columns, gel spheres, gel films, etc. [13]. Meanwhile, supercritical drying, freeze-drying, and evaporative drying can all be used to obtain porous solid aerogels [14].

**Figure 1:**
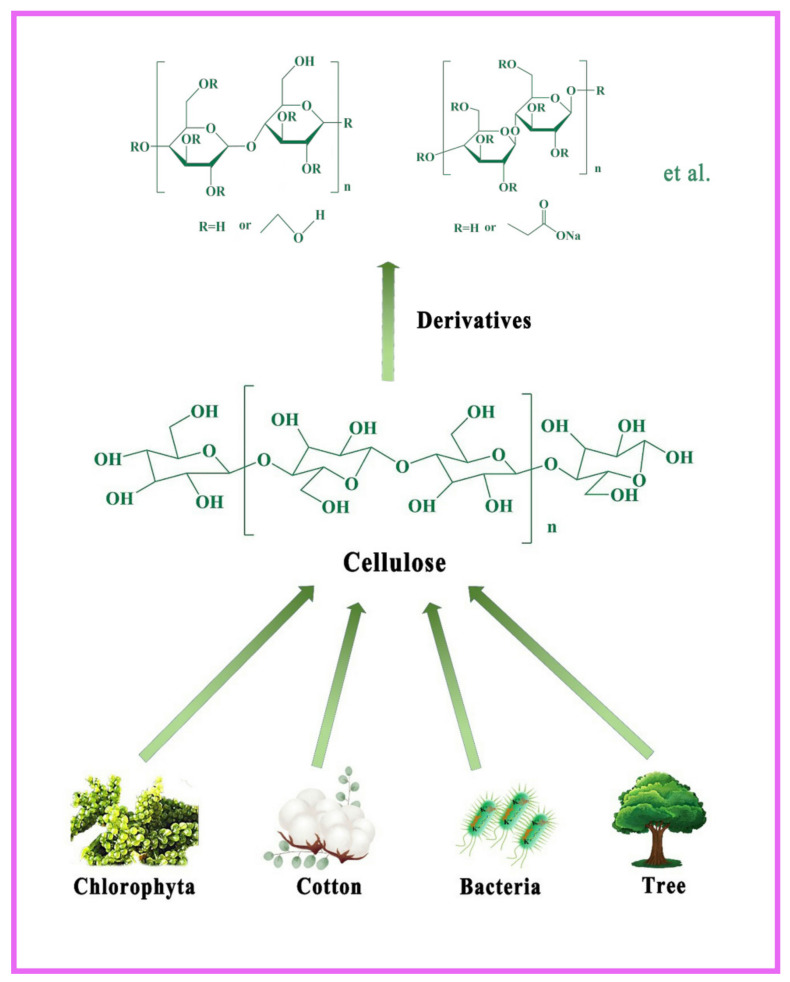
Molecular structure of cellulose and its origin in nature.

With the increasing emphasis on the development and utilization of renewable resources, bio-based polymers have received a lot of attention from researchers. The biocompatibility and degradability of bio-based hydrogels have led to their application in agriculture [15], medical [16], and environmental fields [17]. Cellulose is a linear polymer composed of many D-glucopyranose units interconnected by β-glycosidic bonds. It is one of the most widely distributed polysaccharides in nature, and is present in several plants, bacteria, and algae such as cotton, rice straw, trees, and Chlorophyta. [18,19,20] (Figure 1). The exposed hydroxyl groups of cellulose can undergo more chemical reactions, providing the possibility of preparing cellulose derivatives, optimizing their disadvantages such as water solubility and poor mechanical properties [21]. With their non-toxicity, and easy degradability, hydrogels of cellulose and its descendants can be used in agriculture (storage and continuous release of water and fertilizers [22]), water treatment (adsorption of heavy metal ions [23], desalination of seawater [24], photocatalytic degradation of organic pollutants [25], or photocatalytic sterilization [26]), and biomedicine (drug delivery [27], wound dressings [28], tissue engineering [29], health care hygiene [30], and smart materials [31]), among other areas.

Cellulose hydrogels are cited for use in both the water treatment [32] and antimicrobial fields [33], emphasizing their adsorption properties as well as their photocatalytic properties. This paper mainly summarizes the properties, preparation methods, and classification of cellulose-based hydrogel materials and their different applications in the direction of photocatalysis over the past 15 years, providing a good basis for future development.

## 2. Characteristics of Cellulose-Based Hydrogel Photocatalytic Composites

Cellulose hydrogels as semiconductor carriers have three main characteristics: high adsorption, dispersibility, and morphological auxiliary. The adjustment of these characteristics can change the structural features of the semiconductor, which in turn, can effectively improve photocatalytic efficiency.

### 2.1. High Adsorption

Cellulose molecules form intramolecular and intermolecular hydrogen bonds, and the molecular chains are coiled to form highly crystalline fibers that are more difficult to dissolve. At the same time, hydroxyl groups are blocked within, which affects their adsorption properties for water, oil, and heavy metal ions [34]. In practical applications, cellulose is chemically modified (oxidation, esterification, grafting, etc.) by introducing specific groups to avoid its insolubility and enhance its adsorption properties [35,36]. Therefore, cellulose derivatives are common materials for preparing cellulose-based hydrogels, specifically: hydroxy cellulose (HEC), carboxy cellulose (CMC), and amino cellulose. Han et al. prepared titanium dioxide hydrogel cages using HEC and CMC. The hydrogel cages showed good adsorption performance: within 5 min, the hydrogel cages adsorbed 43% more dye than titanium dioxide nanoparticles, which greatly enhanced the photocatalytic performance of the composites [37]. Adsorption is an essential part of the photocatalytic link, and a high stirring speed is usually used to reduce the mass transfer resistance. Hydroxy cellulose and carboxy cellulose hydrogel photocatalytic composites enhance the adsorption of dyes in wastewater through electrostatic interaction, which in turn promotes photocatalytic reaction activity. In short, a synergistic adsorption–photocatalytic system was constructed to enhance the photocatalytic effect [38,39]. Amino cellulose has an amino group at the end, which is similar to the structure of chitosan. The introduction of the amino group makes it very soluble, film-forming, and adsorbent of heavy metal ions, yielding good prospects for biological applications such as wound dressing, immunofluorescence, and drug release [40,41,42]. However, the complex synthesis process of amino cellulose and the poor selectivity and economy of the synthesis process hinders the production and limits the application of amino cellulose. No research on hydrogel-type photocatalytic composites has been done in photocatalysis.

### 2.2. Dispersibility

**Figure 2:**
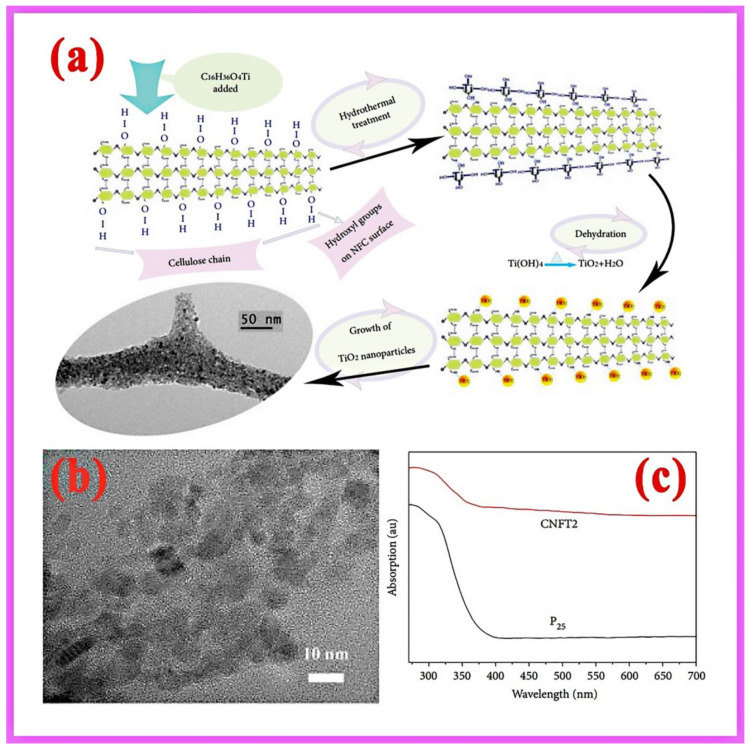
(**a**) Synthesis mechanism of cellulose nanotitanium dioxide aerogel (CNFT); (**b**) TEM micrograph of CNFT2; (**c**) UV-Vis spectra of P25 and CNFT2 [44].

Nanophotocatalysts have high specific surface energy and are thermodynamically unstable systems. The nanoparticles agglomerate to form soft and hard agglomerates due to van der Waals and Coulomb forces between the particles during preparation or post-processing, affecting the adequate performance of their photocatalytic properties [43]. Therefore, cellulose can be a suitable carrier for improving the dispersion of nanophotocatalysts to expose more active sites to capture light or change the semiconductor bandgap to participate in the reaction. The atoms and ions of semiconductors are anchored by functional groups in cellulose through chemical or hydrogen bonding. For example, based on the chemical interaction between Zn^2+^ and COO^−^ of ZnO, Zn^2+^ was adsorbed in floatable carboxy methyl cellulose/polyphenyl amide hydrogel (PAM/CMC/DDM), which enabled the practical separation of heavy metal ions in sewage. Subsequently, PAM/CMC/DDM-ZnO photocatalytic composite was obtained by processing Zn^2+^ into ZnO nanoparticles using an in situ precipitation method. The nanoparticles in this composite avoided agglomeration during the preparation processes, creating a highly efficiently degradation of the dye under visible light [25]. The hydroxyl groups of cellulose can form strong hydrogen-bonding interactions with titanium dioxide nanoparticles, which can be made to adhere to the cellulose surface using a hydrothermal method to obtain cellulose nanofiber/titanium dioxide (P25) aerogel (CNFT) (Figure 2a). The P25 in CNFT2 is uniformly dispersed, and the transmission electron micrographs show that its average diameter is around 6.8 nm (Figure 2b). Moreover, the spectral red-shift of the composites was obtained by UV-vis diffuse reflection (Figure 2c), narrowing the bandgap of P25 and favoring the photocatalytic reaction [44].

### 2.3. Morphological Adjuvants

Usually, controlling the photocatalyst morphology is also a meaningful means of enhancing the catalyst activity. The structure, specific surface area, crystal shape, and crystal defect of photocatalyst are the factors that affect the separation of photogenerated electrons from holes [45]. In contrast to the existing morphology-modulating auxiliaries, cellulose-based hydrogels have the advantages of being green, simple, and efficient.

Cellulose hydrogels can be used as reactors. Qin et al. successfully prepared flower-like ZnO nanoparticles with a crystalline form of hexagonal fibrous zincite from sodium hydroxide and zinc acetate. In this study, there is a chemical bonding between the hydrogel reactor and sodium hydroxide and water, which makes Zn^2+^ and OH^−^ slowly combine into [Zn(OH)_4_]^2−^ ions in the three-dimensional pores. Finally, the nanoflowers are generated by dehydration, induction into nanosheets, and self-assembly, yielding homogeneous size of the flower-like ZnO nanosheets in cellulose hydrogel pores and a high surface area (39.18 m^2^/g) after calcination. It accelerated a decrease in rhodamine B concentration under UV light [46].

Cellulose hydrogels act as green capping agents to guide the semiconductor shape change. As a typical example, Sabbaghan et al. selectively prepared cellulose oxide gel membranes of different shapes (NFC/Cu_2_O) (spherical, cubic, and truncated cubic) using the reduced end groups of cellulose for Cu^2+^ ion binding. The bandgap of the NFC/Cu_2_O films with different shapes was shown to be in the range of 2.02–2.25 eV by inspection, and this optical property opens up new applications for cellulose gel films [47].

## 3. Preparation of Cellulose-Based Hydrogel Photocatalytic Composites

There is a cross-linking of the hydroxyl, acetyl, and carboxyl functional groups of cellulose and a loading of photocatalyst to obtain hydrogel photocatalytic composites. Standard cross-linking methods can be categorized as physical, chemical, and radiation cross-linking methods (Table 1).

### 3.1. Physical Cross-Linking Method

The physical cross-linking method is mainly based on hydrogen bonding, crystallization, and van der Waals forces to obtain hydrogels with a three-dimensional network structure [48]. Its operation is simple, but the reversible connection between the chains means that heat will return the gel the solution state [49]. Su et al. heated cellulose, carrageenan, and titanium dioxide in 1-ethyl-3-methylimidazole acetate solution, then cooled and washed it to obtain hydrogel photocatalytic membranes by hydrogen bonding [50].

### 3.2. Chemical Cross-Linking Method

The chemical cross-linking method uses covalent bonds between molecules to form hydrogels with desirable stable structures and mechanical strength. This method is accessible using a broad range of monomers and mild conditions, usually with cross-linking agents such as acrylic acid, polyethylene glycol, ammonium persulfate, polyethyleneimine, etc. Su et al. used ammonium persulfate to generate free radicals in cellulose, which cross-linked with acrylic acid (AA) and acrylamide (AM). Meanwhile, the Cu source was added and freeze-dried to gain Cu_2_O/cellulose-based aerogels. The adsorption of large amounts of molecular oxygen at the surface of aerogels promotes the separation of Cu_2_O photoelectrons and holes, thus enhancing the catalytic activity [51].

### 3.3. Radiation Cross-Linking Method

Radiation technology uses the interaction between rays, accelerated electrons, ions, and substances to ionize and excite in order to produce free radicals, and initiate cross-linking reactions [52]. The radiation cross linking method has the advantages of simple operation, room temperature reaction, high efficiency, and green and non-polluting properties when compared to the above methods [53]. Liu and his colleagues used the electron beam radiation method to develop poly-N-isopropyl acrylamide/highly substituted hydroxypropyl cellulose/carbon nitride (NIPAAm/HHPC/g-C_3_N_4_) intelligent hydrogels, which is a thermally driven property photocatalyst. The high specific surface area, porosity, and large specific surface area of this hydrogel enhance the contact of rhodamine (RhB) dyes. The networked three-dimensional structure of the hydrogel effectively adsorbed RhB dye ions, achieving the combination of adsorption and photocatalysis [54].

## 4. Classification of Cellulose-Based Hydrogel Photocatalytic Materials

Based on the current research results, the cellulose-based hydrogel photocatalytic materials can be classified into metal oxide semiconductor composites, metal sulfide (chloride) semiconductor composites, and organic semiconductor composites according to the types of photocatalysts (Table 1).

### 4.1. Metal Oxide Semiconductor Composites

Currently, most of the metal oxide semiconductors applied in cellulose hydrogels are titanium dioxide, due to their stable nature, non-toxicity, and cheapness [55]. Earlier, researchers considered that toxic cross linking agents (dialkyl sulfone) would remain in the synthesis of cellulose hydrogels, causing water contamination [56]. Therefore, a new highly absorbent and biodegradable hydrogel was synthesized using sodium carboxymethyl cellulose (CMCNa), hydroxyethyl cellulose (HEC), and titanium dioxide nanoparticles (TiO_2_). This hydrogel was entirely degraded by dialkyl sulfone under 5 h of light [57]. Subsequently, several hydrogels with unique properties have been developed. For example, high-temperature resistant cotton fiber aerogel [58]; α-cellulose hydrogel as TiO_2_ in situ reactors with excellent strength and good toughness [59]; high stiffness titanium dioxide/polyacrylamide/chitin oxide nanofiber hydrogel (TiO_2_-TOCNs-PAM), its compressive strength at 70% strain is 1.46 MPa, tensile stress is 316 kPa, tensile strain is 310%, and toughness is 47.25 kJ/m^3^ [60]; multifunctional flexible bacterial cellulose gel film with self-cleaning, photocatalytic, and UV protection properties [61]; and cellulose nanofiber aerogels loaded with TiO_2_, with good adsorption properties, high photocatalytic degradation, low density, and easy recycling [62].

ZnO nanoparticles and cuprous oxide are also materials of interest to researchers. Hasanpour et al. prepared six different shapes of cellulose/ZnO (CA/ZnO) heterogeneous aerogels using microcrystalline cellulose (MCC) and zinc nitrate hexahydrate as the primary raw materials by means of hydrothermal, sol-gel, and impregnation methods. Among these, the highest degradation rate of MO was 94.78% for the CA/ZnO heterogeneous aerogel in plate shape [63]. However, iron trioxide (α-Fe_2_O_3_), sodium trititanate (Na_2_Ti_3_O_7_), silver phosphate (Ag_3_PO_4_), and bismuth vanadate (BiVO_4_) semiconductors are relatively rare composites combined with cellulose hydrogels due to their high price or complexity of preparation [64,65,66,67,68].

### 4.2. Metal Sulfide (Chloride) Semiconductor Composites

Metal sulfides have a narrower bandgap compared to metal oxides. Currently, cadmium sulfide (CdS) and molybdenum sulfide (MoS_2_) are mostly studied in cellulose-based hydrogel photocatalytic materials [69]. CdS crystals are one of the best visible light-reactive photocatalysts. Its forbidden bandwidth is 2.4 eV [70]. CdS nanoparticles are combined with cellulose to form hydrogel composites, and their strong adsorption ability on MB molecules indirectly improves photocatalytic activity [23,71]. Cadmium sulfide solid solution (Cd_x_Zn_1-x_S) can be used to improve the optical properties of the catalyst by modulating the elemental composition. Wu et al. used in situ chemistry to embed Cd_x_Zn_1-x_S particles with a dimension of about 3 nm into carboxymethyl cellulose hydrogels. Among them, the maximum hydrogen yield of Cd_0.2_Zn_0.8_S gel composites was 1762.5 µmol g^−1^ h^−1^. This is 104 times the hydrogen production rate of pure cadmium sulfide. This hydrogel photocatalytic complex is stable and easily recyclable, meeting the criteria for green hydrogen production [72]. The surface of noble metal nanoparticles can absorb visible light and has a surface plasmon effect [73]. On the path of surface plasmon photocatalyst exploration, Ag/AgCl has been the most studied by scientists. Heidarpour et al. wrapped Ag/AgCl in Al(III) and Fe(III) crosslinked cellulose hydrogel beads (Ag/AgCl@Al-CMC and Ag/AgCl@Fe-CMC, respectively). Experimental tests showed that the gel beads have good photocatalytic properties. The diverse cases on the photocatalytic performance was also explored, and the photocatalytic reaction rate constants are shown in Table 2 [74].

### 4.3. Organic Semiconductor Composites

Graphitic phase carbon nitride (g-C_3_N_4_), graphene oxide (GO), and organic metal frameworks are representative materials for organic conjugated semiconductors. Graphitic-phase carbon nitride is a layered material consisting of triazine and tri-s-triazine rings as basic units [75]. It is of interest because of its advantages, such as being non-toxic, cheap, and responsive in visible light. However, the disadvantages of carbon nitride, such as a small specific surface area, easy polymerization, and few active sites, affect its photocatalytic performance [76]. Combining it with cellulose to form aerogel photocatalytic materials can expand the specific surface area and upgrade carrier separation, thus improving the photocatalytic ability [77,78,79]. GO has a large specific surface area compared to g-C_3_N_4_. Its large number of hydroxyl and carboxyl groups can be used for adsorption. However, GO is soluble in water and difficult to use as an adsorbent [80]. Combining it with MCC and polyaniline (PANI) perpetuates the adsorption performance and achieves sound synergistic adsorption-photocatalytic degradation [81]. Metal-organic frameworks (MOFs) are composed of metal units and organic ligands combined in a framework by coordination to form an open network with high porosity, a stable network, and a massive surface area. MIL-100(Fe) is merged with CMC and cyclodextrin to form a hydrogel with catalytic and water fixation capabilities. It has a good hydrophilicity, with a swelling rate of 363%, which allows it to be used in environmental applications [82].

## 5. Application of Cellulose-Based Hydrogel Photocatalytic Materials

The advantages of cellulose-based hydrogel photocatalytic composites and photocatalysts and hydrogels are combined to promote cellulose with unique properties for different applications. In this section, the latest applications of cellulose-based hydrogel photocatalysts in wastewater treatment and energy will be briefly outlined.

### 5.1. Wastewater Treatment

The insolubility and hydrophilicity of most types of cellulose make cellulose-based hydrogel photocatalytic composites widely used in wastewater treatment.

#### 5.1.1. Removal of Dyes and Heavy Metal Ions

The degradation of dyes and heavy metals are the two most frequent methods used to evaluate photocatalytic performance. Every year, printing and dyeing processes produce hundreds of millions of tons of highly concentrated wastewater containing different types of dyes, in addition to heavy metals, acids, and bases, causing severe environmental problems [83,84]. The dyes and heavy metals commonly used for photocatalytic degradation are rhodamine B [85,86], methyl orange [87,88], methylene blue [89], carmine [90], and hexavalent chromium ions [91]. Cellulose hydrogels carry functional groups that enhance the adsorption and induce the photocatalytic degradation of dyes [92,93]. Table 3 lists the degradation efficiency of methyl orange (MO) by different photocatalytic materials.

**Figure 3:**
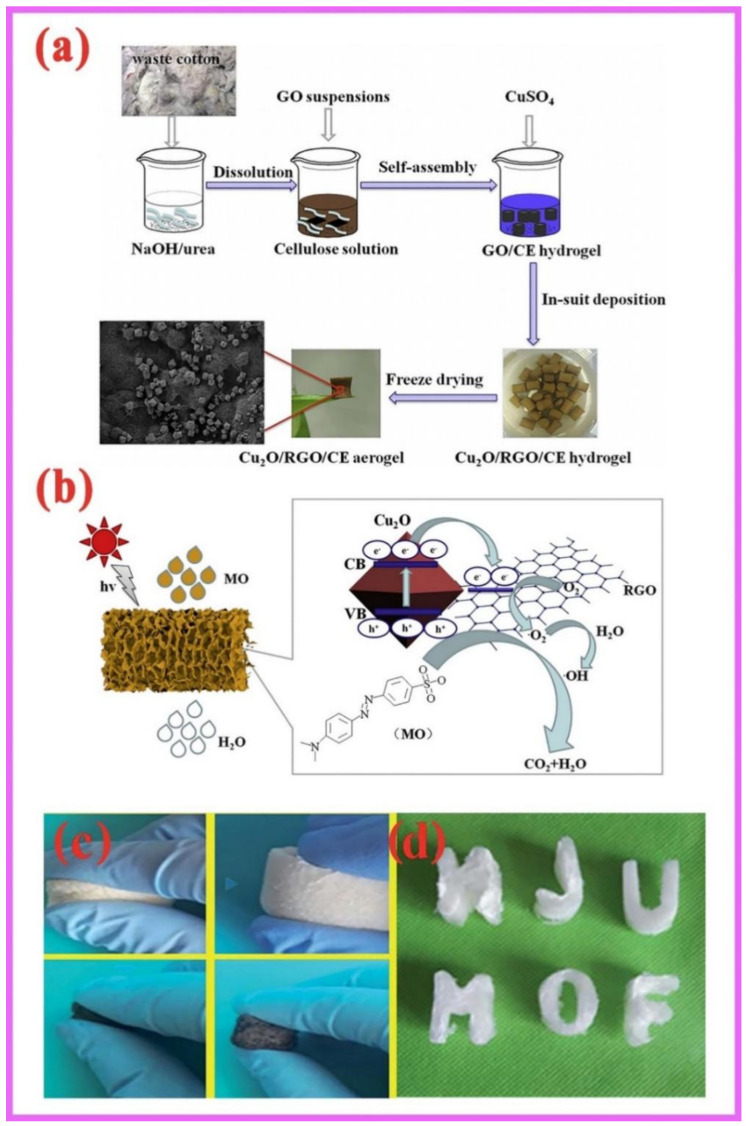
(**a**) Synthesis process of Cu@Cu_2_O/RGO/CE hybrid catalysts; (**b**) Mechanism diagram of Cu@Cu_2_O/RGO/CE photocatalytic degradation of MO; (**c**) Elasticity test map; and (**d**) plasticity of BMFAs [94,95].

Two representative cases are presented in particular. Du et al. synthesized layered stomatal Cu/doped Cu_2_O/reduced graphene oxide/cellulose (Cu@Cu_2_O/RGO/CE) catalytic materials using the in situ deposition method (Figure 3a). It had a high photocatalytic performance for the degradation of MO in visible light. A reasonable photocatalytic degradation diagram was obtained by EPR tests showing that hydroxyl radicals and superoxide radicals take effect in the photocatalytic process (Figure 3b) [94]. NH_2_-MIL-88B(Fe) (NM88) and g-C_3_N_4_ loaded onto aerogels, combined with natural cellulose and polyacrylonitrile fibers (BMFAs), achieved a 99% reduction of Cr(VI) within 20 min. At the same time, this composite has excellent memory properties and shape deformability (Figure 3c) [95].

#### 5.1.2. Degradation of Antibiotics

Antibiotics are remarkably effective in treating infectious diseases and are in high demand in the livestock and aquaculture industries. However, residual antibiotics can also have severe ecological and public health impacts [96]. Therefore, the problem of antibiotic reprocessing is among the urgent issues to be addressed. Currently, efficient, mild, and non-polluting photocatalytic technology shows good prospects for degrading antibiotic wastewater. Tetracycline, as a spectral antibiotic, has received significant attention from researchers. Recent literature reported that the combination of photocatalyst and cellulose to form an open porous three-dimensional structure and high specific surface area enhanced its adsorption, thus improving the degradation efficiency [97]. The high mechanical strength of hydrogels allows the reusability of the composites [98].

#### 5.1.3. Antibacterial Properties

After an assessment by the World Health Organization (WHO), the following information was obtained. In developing countries, 80% of diseases originate from water sources contaminated with pathogenic microorganisms, including fungi, bacteria, and viruses [99]. These microorganisms can cause diseases such as diarrhea, typhoid, and pneumonia. Photocatalysis has received attention from researchers as an effective and inexpensive method for sterilization. Zhang et al. synthesized multifunctional cellulose/TiO_2_/β-CD hydrogels with extreme photocatalytic antibacterial properties and drug release capacity. Their excellent photocatalytic antibacterial activity was verified by the inhibition circle method under dark and light conditions [100].

### 5.2. Energy

#### 5.2.1. Hydrogen Energy

In today’s society, the development of green energy plays a vital role in economic development and human living standards. Hydrogen energy with high calorific value and no secondary pollution is becoming a hot spot for research. In contrast to electrochemical hydrogen generation [101] and anaerobic microbial fermentation [102], photocatalytic hydrogen generation is essential for the development of hydrogen energy by converting sunlight into hydrogen energy using water as a raw material. Kang et al. used an in situ method to combine nanoscale CdS and CMC, while doping with trace amounts of Pt, to produce highly efficient hydrogen-producing photocatalytic hydrogels, with a hydrogen generation efficiency of 1365 μmol h^−1^g^−1^. The cellulose hydrogel enabled a better dispersion of CdS nanoparticles and avoided the secondary contamination of nanoparticles, which has practical implications for the development of hydrogen energy [103].

#### 5.2.2. Food Packaging

Food packaging bags mainly include two types: plastic and paper bags. Plastic bags make up the bulk of packaging materials, prepared by the polymerization of ethylene in petroleum cracking; these are not easily degradable, and doing so will cause secondary pollution. At the same time, due to energy constraints, the transformation of plastic packaging bags is imminent. Cellulose is easily accessible and biodegradable, laying the foundation for the development of green packaging materials. Xie et al. prepared a cellulose gel film containing zinc oxide nanoparticles on the surface by chemical cross-linking and hydrothermal methods. This food packaging film has specific mechanical properties to block oxygen and water vapor and ensure the freshness of food. At the same time, it has some antibacterial effects under both dark and UV light irradiation conditions. Under UV light, the bacteria were inactivated more efficiently by synergistic photocatalytic oxidation and mechanical rupture [104].

## 6. Conclusions and Prospect

Cellulose is widely distributed in nature, has the largest reserves, and is easily degradable. Therefore, the development of functional cellulose materials is of significant meaning to the progress of green chemistry and the reduction of dependence on fossil resources. The functional properties of cellulose, including hydrophilicity and good biocompatibility, make it a suitable carrier for photocatalysts. In this paper, we review the research progress of cellulose-based hydrogel photocatalytic materials, detailing the properties, preparation methods, classification, and applications of the composites in the environmental and energy fields. Combined with the above literature, it is concluded that cellulose hydrogels as carriers exhibit the following main advantages. First, the three-dimensional network of cellulose hydrogels increases the specific surface area, which dramatically improves the adsorption performance of composites. Second, the negative ions on cellulose can bind metal cations, which can immobilize the photocatalyst on the surface and improve the dispersibility of nanoparticles. Finally, cellulose can improve the carrier separation efficiency.

Although the research on cellulose-based hydrogel photocatalytic composites continues to progress, there are still some issues of concern: (1) From the preparation method of composites, radiation preparation has the advantages of green qualities and high efficiency, but relatively little research has been reported on this method. (2) Whether or not the mechanical strength of a hydrogel carrier will be affected during the photocatalytic cycle. Therefore, further research is needed. (3) Most of the applications of the composites are concentrated in the environmental field and very few are developed in the energy field. We have not seen any research in the medical fields, such as photodynamic therapy. Therefore, future research can focus on this area and fully expand its biocompatibility to enable its application in the medical field.

## Figures and Tables

**Table 1:** Classification of cellulose-based hydrogel photocatalytic composites.

Categories	Photocatalysts	Hydrogel Materials	Characteristics	Preparation Methods	Specific Surface Area/m^2^g^−1^	References
Metal Oxide Semiconductor Composites	ZnO	PAM/CMC/DDM	Suspended hydrogels, adsorb heavy metal ions, and degrade dyes efficiently	Mechanical foaming and in situ polymerization	-	Zhao et al. 2021
		Cellulose	Dispersion framework for nanomaterials	Physical crosslinking	-	Jiao et al. 2018
		Bamboo fiber	High specific surface area	Chemical crosslinking	39.18	Qin et al. 2017
	Cu_2_O	Cellulose/AA/AM	High adsorption	Chemical crosslinking	89.56	Su et al. 2017
	TiO_2_	CMCNa/HEC	Superabsorbent, biodegradable, and photocatalytic degradation crosslinker	Chemical crosslinking	-	Marcı et al. 2006
		Cotton cellulose	High temperature resistant	Physical crosslinking	6.10	Melone et al. 2013
		α-Cellulose	TiO_2_ in situ generators, excellent strength and good toughness	Chemical crosslinking	550	Wang et al. 2017
		TOCNs/PAM	Super-tough	Chemical crosslinking	-	Yue et al. 2020
		BC	Self-cleaning, antibacterial, and UV shielding	Chemical crosslinking	-	Rahman et al. 2021
		CNFs	Good adsorption, photocatalytic degradation ability, low density, and easy recovery	Chemical crosslinking	330	Li et al. 2021
	Na_2_Ti_3_O_7_	Sisal cellulose	High specific surface area	Physical crosslinking	248.93	Liu et al. 2021
Metal sulfide (chloride) semiconductor composites	MoS_2_	BC	Bifunctional adsorbent/photocatalyst membranes	Chemical crosslinking	137	Ferreira-Neto et al. 2022
	CdS	Straw cellulose	Green recyclable	Chemical crosslinking	-	Qian et al. 2020
	Cd_x_Zn_1-x_S	CMC	High yield of hydrogen, good stability, easy recovery	Chemical crosslinking	-	Wu et al. 2018
	Ag/AgCl	CMC	Hydrogel beads, photocatalytic degradation of RhB	Chemical crosslinking	-	Heidarpour et al. 2020
Organic semiconductor composites	g-C_3_N_4_	Polyester fiber/cotton wool	High specific surface area, impact resistant	Chemical crosslinking	-	Chen et al. 2019
		Cotton linter	Enhanced carrier separation	-	-	Bai et al. 2019; Yao et al. 2019
		CMC/β-Cyclodextrin				
	GO	MCC	Adsorption–photocatalytic synergy	Chemical crosslinking	48.6	Liu et al. 2021
	MIL-100(Fe)	CMC/β-Cyclodextrin	Good water retainability	Chemical crosslinking	-	Zhang et al. 2021

**Table 2:** Photocatalytic rate constants of Ag/AgCl@Al-CMC and Ag/AgCl@Fe-CMC under different conditions ((copied from Reference [74]).

	Ag/AgCl@Ag-CMC				AgCl@Fe-CMC			
Catalyst Dosage	1 (g/L)	2 (g/L)	4 (g/L)	6 (g/L)	1 (g/L)	2 (g/L)	4 (g/L)	6 (g/L)
**K_app_**	0.0101	0.0223	0.0517	0.0711	0.0073	0.0152	0.0304	0.0395
**R^2^**	0.98	0.99	0.95	0.98	0.98	0.99	0.99	0.99
**RhB concentration**	10 (ppm)	15 (ppm)	20 (ppm)	25 (ppm)	10 (ppm)	15 (ppm)	20 (ppm)	25 (ppm)
**K_app_**	0.0517	0.0318	0.0233	0.0141	0.0304	0.0224	0.0170	0.0103
**R^2^**	0.95	0.99	0.97	0.99	0.99	0.99	0.98	0.99
**pH**	4	7	9		4	7	9	
**K_app_**	0.0295	0.0517	0.0673		0.0198	0.0304	0.0352	
**R^2^**	0.99	0.95	0.99		0.99	0.99	0.95	

**Table 3:** Degradation efficiency of MO by different photocatalytic materials.

Dye	Catalysts	Dye Concentration (mg/L)	Time (min)	Degradation (%)	References
MO	TiO_2_-TOCNs-PAM	10	90	97.3	Yue et al. 2020
	CA/ZnO	20	120	94.78	Hasanpour et al. 2021
	g-C_3_N_4_ Cellulose aerogel	20	180	99	Ma et al. 2021
	Ag@AgCl-contained cellulose hydrogel	10	70	93	Tang et al. 2018
	Cu_2_O/TiO_2_/CNF/rGH	20	120	85.62	Zheng et al. 2022
	Cu@Cu_2_O/RGO/cellulose hybrid aerogel	10	120	92.8	Du et al. 2019
